# Gentamicin-Coated Tibia Nail in Fractures and Nonunion to Reduce Fracture-Related Infections: A Systematic Review

**DOI:** 10.3390/molecules25225471

**Published:** 2020-11-23

**Authors:** Daniele De Meo, Federico M. Cannari, Luisa Petriello, Pietro Persiani, Ciro Villani

**Affiliations:** 1 Orthopaedic and Traumatology Department, Policlinico Umberto I Hospital-Sapienza, University of Rome, Piazzale A. Moro, 3, 00185 Rome, Italy; luisa.petriello@gmail.com (L.P.); ppersiani@me.com (P.P.); ciro.villani@uniroma1.it (C.V.); 2 M.I.T.O. Group (Infectious Diseases in Traumatology and Orthopedics Surgery), Policlinico Umberto I Hospital, Viale del Policlinico, 155,00161 Rome, Italy; 3 Orthopaedic and Traumatology Department, Tor Vergata University, Via Cracovia, 50,00133 Rome, Italy; federico.cannari@gmail.com

**Keywords:** coating, intramedullary nailing, tibia fracture, nonunion, complication, fracture-related infection, osteomyelitis, gentamicin

## Abstract

The incidence of a fracture-related infection (FRI) can reach 30% of open tibia fractures (OTF). The use of antibiotic-coated implants is one of the newest strategies to reduce the risk of infection in orthopedic surgery. The aim of this study was to investigate the efficacy and safety of a gentamicin-coated tibia nail in primary fracture fixation (FF) and revision surgery (RS) of nonunion cases in terms of FRI incidence. We conducted a systematic review according to the PRISMA checklist on Pub-Med, Cochrane, and EMBASE. Of the 32 studies, 8 were included, for a total of 203 patients treated: 114 were FF cases (63% open fractures) and 89 were RS cases, of which 43% were infected nonunion. In the FF group, four FRI were found (3.8%): three OTF (Gustilo-Anderson III) and one closed fracture; bone healing was achieved in 94% of these cases. There were four relapses of infection and one new onset in the RS group; bone healing occurred in 88% of these cases. No side effects were found. There were no significant differences in terms of FRI, nonunion, and healing between the two groups. Gentamicin-coated tibia nail is an effective therapeutic option in the prophylaxis of high-risk fracture infections and in complex nonunion cases.

## 1. Introduction

Tibia shaft fractures are the most common long-bone fractures among adults and children [1]. They have a deep socio-economic impact, accounting for approximately 26 fractures per 100,000 and 569,000 hospital days per year [1,2]. Men have a three times higher risk of fracture than women. Incidence increases in young adults who suffer high-energy trauma or in elderly people who suffer low-energy trauma directed towards poor quality bone tissue due to osteoporosis [3]. Incidence of nonunion in the general population is approximately 12% of all tibia fractures, and in open fractures this can raise up to 23% [4]. Open tibia fractures account for two per 1000 injuries [5]. A mention should be made of elderly people, as a substantial difference in the fracture pattern can be observed; moreover, the rate of open fractures in these patients can be as high as 30%, of which 10% are nonunion and 17% are malunion [6]. A fracture-related infection (FRI) is a serious and complex consequence of a tibia fracture: the economic, financial, and health costs can be enormous. Health care costs can in fact increase by about 6.5 times [7]. In total, 64% of all FRI are located in the tibia, making it the area with the highest risk of infection [8]. Open tibia fractures have a risk of developing a superficial or deep infection ranging from 6% to 33%, depending on the type of fracture and degree of bone exposure [9]. Currently, systemic antibiotic prophylaxis associated with good surgical management is the gold standard in the prevention and treatment of the aforementioned FRI [10]. There is an inherent limit to systemic prophylaxis as bacteria that typically develop FRI tend to form a biofilm at the level of the implants. This biofilm is particularly difficult to attack by systemic means because of its biologically active matrix that links to metallic surfaces and shields the bacteria from antibiotics and chemical agents [11]. To overcome this limit, antibiotic-coated implants have been proposed to increase the local antibiotic concentration without systemic side effects [12]. The ETN PROtect™ (DePuy Synthes Companies, Zuchwil, Switzerland) is a tibial coated nail that consists of an alloy of titanium, aluminum, and niobium and is coated with an absorbable poly (d, l-lactide) (PDLLA) matrix with gentamicin sulfate incorporated. Throughout a proprietary process the entire surface of the nail is coated homogeneously [13,14]. The characteristics of PDLLA and gentamicin have not been altered during the production and sterilization processes and they also did not interfere with each other [15]. Depending on the size of the implant, the amount of gentamicin is between 10 and 50 mg. About 40% of the total amount of gentamicin is released in the first hour following implantation; roughly 70% is then released after 24 h and 80% after 48 h [16]. In fact, as the gentamicin is embedded into a PDLLA matrix, the initial burst release is followed by a secondary prolonged release due to the constant degradation of the newly exposed polymer [15]. 

Manufacturers recommend this product for exposed fractures (Gustilo Anderson—GA—Classification I to III), revision surgeries after infection, after polytrauma and in immunosuppressed patients [17]. The purpose of this study was to summarize the current evidence about the clinical use, the efficacy, and the safety profile of this gentamicin-coated tibial nail.

## 2. Results

**Figure 1:**
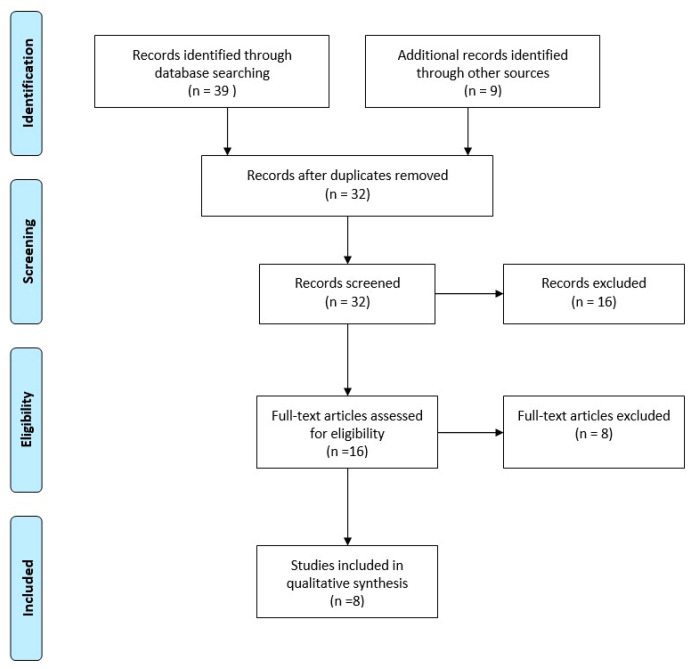
PRISMA flow diagram of the systematic review.

The literature search, excluding duplicates, resulted in 32 records (Figure 1). After a full text review, eight studies were included for final analysis (Table 1) [16,18,19,20,21,22,23,24]. According to Oxford’s Level of Evidence, there was one study at level II, one study at level III, and six studies at level IV. Four studies had prospective study-design; the others were retrospective. Among them, two case reports were retrieved and included in the review [18,19]. No studies reported a control population treated with a nail without gentamicin coating, except for Pinto et al., which performed a prospective study with randomization by alternate allocation of the patients [24]. Out of these studies, 203 patients were retrieved; the median age was 45 ± 13 years. Of these patients, 53 were female (28.04%). Only three studies reported the presence of comorbidity [16,21,22]; in those series (n = 154), there were 44 smokers (28.57%), 22 patients addicted to alcohol (14.29%), and 9 patients affected by diabetes (5.84%). There were 39 polytraumatized cases (25.49%) out of 153 patients taken from all the studies that report this information [16,18,19,20,21,23]. Patients were subdivided into two groups: the fixation of recent fractures (within 4 weeks from trauma) group (FF—Fracture fixation) and the revision cases (previously treated with other type of implant) group (RS—Revision surgery).

### 2.1. Fracture Fixation Group

One hundred and fourteen patients had a recent fracture treated with coated nail, reported in six studies [16,18,19,20,21,24]. Among them were 42 closed fractures (36.84%). Of the 72 patients with an open fracture, 20 were affected by a GA I, 23 patients sustained a GA II, and 52 cases reported a GA III. All studies except Schmidmaier’s (n = 46) reported if the nail was the first surgery performed or if there was other temporary stabilization: 22 patients underwent an immediate nailing (47.83%). One hundred and five patients (92.1%) were available at last follow-up (FU) with a median of 14 ± 7 months. Among those patients, there were four FRI (3.81%): one patient with a closed fracture and three cases with a GA III open fracture. Three patients sustained a superficial surgical site infection (SSI). There was bone healing evidence in 80 patients (76.19%) and partial healing in 19 patients (18.09%); nonunion occurred in six patients (5.71%). Thirty-three patients underwent further surgical operations: 25 nail dynamizations to achieve maximum bone healing stimuli and eight reoperations. Fuchs and Schmidmaier also reported functional outcomes with an SF-36 Physical of 42.55 and 44.6, respectively, and a SF-36 Mental of 50.45 and 53.1, respectively. Principal features of this group are reported in Table 2.

### 2.2. Revision Surgery Group

Eighty-nine patients underwent revision surgery with the coated nail after a previous tibia fracture, reported in four studies [20,21,22,23]. Among them, there were 64 nonunion cases (71.91%) and 25 patients already infected. Sixty-two patients had a previous open fracture; 3 were affected by a GA I, 24 patients sustained a GA II, 28 cases reported a GA III, and 7 patients had an exposed fracture but it was impossible to retrieve more information. Moghaddam and Vicenti reported the use of Reaming Irrigation and Aspiration system and bone grafting in 29 of 32 patients of their series [22,23]. Moreover, Mogghadam also reported the coated nail implantation as a second stage of a Masquelet technique in 12 patients. Seventy-seven patients (86.52%) were available at last FU (median of 14 ± 3 months). Five FRI were reported in this group (6.49%): four were relapses of previously infected patients and one was a new onset in a previously aseptic nonunion patient. Nine patients sustained a superficial SSI (11.69%). Bone healing occurred in 64 patients (83.12%), and partial healing occurred in four patients (5.19%); relapse of nonunion happened in nine cases (11.69%). Further surgical operation was needed in six patients (7.79%). The principal features of this group are reported in Table 3.

### 2.3. Statistical Analysis

Statistical analysis was performed to highlight the differences between these two groups and between specific types of features compared to their outcome. No significant statistical differences were found in incidence of FRI (*p* = 0.0642), bone healing (*p* = 0.2745), and nonunion (*p* = 0.1773) between FF and RS groups. In the FF group, there was no statistical difference between open and closed fractures in terms of FRI incidence (*p* = 1.000). In the RS group, there was a statistical difference between FRI that occurred in nonunion patients and previously infected patients (*p* = 0.0208).

## 3. Discussion

### 3.1. Efficacy

The prophylactic efficacy of coated nails in the FF group is disputed; there is not a sharp reduction of FRI incidence if compared to other series treated with non-coated nails [25]. However, going further into detail, the incidence of FRI in open fractures seems to lower if a coated nail is used. In fact, Pinto et al. showed a statistically significant difference (*p* = 0.031) in their randomized study in which FRI was higher in the control group [24]. This is the only trial in the literature that compares coated and non-coated tibia nails. These results should be supported by larger randomized studies comparing those treatments in a more homogeneous population. 

According to the Swedish fracture register, the rate of infection was similar to this systematic review (3.4%, 11/320), while nonunion was higher (8.1%, 26/320); malunion was 3.4% (11/320) and implant failure was 3.7% (12/320). From their data, fracture healing rate in tibial fracture treated with intramedullary nail is 81.25% [26]. 

Local antibacterial strategies have been extensively studied to help surgeons in the prevention and treatment of biofilm-related infections. At the time of writing, the only coatings available on the market for clinical uses are: preloaded gentamicin-coated nail, antibiotic-loaded hydrogel, silver-coated prosthesis, and iodine-coated implants. Antibiotic-loaded hydrogel has been studied both in joint replacement surgery [27] and in trauma surgery [28]; it has been proven to be effective and safe. Compared to the gentamicin PDLLA nail coating, the hydrogel has some advantages. Since the hydrogel must be mixed directly in the operating room with the antibiotic, this allows the choice of antibiotic to be used, being able to combine more of them together. Moreover, the hydrogel can be applied on any fixation device or prosthesis used during surgery. Conversely, it has the disadvantage that it cannot guarantee with certainty a uniform distribution on the implant surface, as it is manually applied to it during the surgery. Future studies should develop and test the application of preloaded PDLLA-antibiotic coatings to other fixation devices. An antibacterial coating without antibiotic release is now facing the market with encouraging results. Silver-coated prostheses are gaining interest and are used especially in tumor surgery. Recent retrospective studies are finding it useful in this specific high risk surgery, but more randomized controlled trials are needed [29]. The use of iodine-coated implants in malignant tumor resection combined with joint replacement may be a promising technique to reduce infection [30]. In the joint replacement field, there are many local strategies that are emerging; in trauma surgery, more instruments are needed, considering the higher risk of infection.

### 3.2. Safety

Gentamicin belongs to the aminoglycoside group; it is active against Gram negatives and the most common bacterial strains found in orthopedic surgeries or following open fractures, above all staphylococcus species. Although its efficacy is high, gentamicin is not commonly used for systemic antibiotic prophylaxis in orthopedic surgery as it has a small therapeutic index and serious dose-dependent side effects [31]. It has been shown that the amount of gentamicin released into circulation by coated nails does not reach systemic toxicity levels [12]. The literature data show how the use of gentamicin-coated nails do not release gentamicin into the systemic circulation above the lowest detectable level of 0.2 mg/dL; therefore, it is safe to say that no severe side effects could be linked to implantation. Furthermore, the use of local gentamicin does not affect bone healing in a negative way [32].

### 3.3. Limits

There are several limitations in this review. First, the relatively low quality of the study and the relatively small population included. Moreover, other limitations were related to the differences in methodological approaches between the studies (i.e., heterogeneity of data, population, treatment protocols, outcome measures, follow-up periods, and so on). An additional limitation was the different type of perioperative antibiotic treatment used in the various studies. Finally, the parameters used during follow-up to detect FRI were not homogeneous; this may have led to a difference in assessment between the studies. In fact, some studies performed a clinical and radiographic follow-up to assess FRI or relapses of previous infections [20,22,23]. Some papers have assessed this endpoint by monitoring C reactive protein, erythrocyte sedimentation rate, hemoglobin, and leukocyte count [16,18,19,21,24]. In suspicious cases, further analyses were performed to confirm FRI.

## 4. Materials and Methods

We conducted a systematic review according to the PRISMA method (Preferred Reporting Items for Systematic Reviews and Meta-Analyses) [33]. Data were collected according to a standardized protocol, in which objectives and inclusion criteria were specified in detail. We searched studies using PubMed, Embase, and Cochrane Library databases. The following keywords were used: “antibiotic coating”, “intramedullary nail”, “tibia fractures”, and “gentamicin-coated nails”. Two reviewers (F.M.C. and D.D.M.) selected potentially relevant records by reviewing titles and abstracts and obtained full copies of the articles. Additionally, all reference lists of the included articles were also reviewed. 

### 4.1. Criteria for Eligibility 

In this systematic review, studies were selected if they followed these criteria: (1) articles were written in English, (2) articles were published after January 2000, (3) all original studies—case report, case series, case control, randomized controlled trials—were considered eligible, and (4) clear data about the onset of FRI, bone healing, and follow-up. Review articles and original studies based on animal experiments were excluded. After eliminating duplicates, two authors (F.M.C. and D.D.M.) separately identified publications for eligibility and resolved any disagreement by consensus. One of the authors (L.P.) reviewed the results and approved the selected studies. The authors then assessed the level of evidence of each study according to the Oxford Center for Evidence-based Medicine (https://www.cebm.net/). 

### 4.2. Data Extraction and Analysis 

After the final selection, data were extracted from the included articles. The following general data were collected: number of patients, presence of control population, sex, age, and comorbidities. According to diagnosis and treatment, patients were subdivided into two groups. The fracture fixation group, which included patients who sustained an open or closed fracture treated with intramedullary-coated nail within 4 weeks of trauma, and the revision surgery group, which comprised patients affected by tibia nonunion, whether infected or not, previously treated with other types of implant. If reported, the degree of Gustilo Anderson classification (GA) for open fractures was recorded. Treatment outcomes were analyzed and complications were identified. The term “facture-related infection” (FRI) was used to include both deep infection and osteomyelitis according to the European Bone and Joint Infection Society consensus definition [34]. Superficial surgical site infections were classified separately. Fracture healing and nonunion were also reported. Further surgeries performed were included as “reoperation”, except for the nail dynamization. The time of follow up was also collected. 

### 4.3. Statistical Analysis 

The statistical analysis was carried out with the software RV 3.4.4 (R Core Team (2018). R: A language and environment for statistical computing. R Foundation for Statistical Computing, Wien, Austria. URL: https://www.r-project.org/). Descriptive statistics were employed where possible. The continuous variables were analyzed with the *t*-test, while the Fisher exact-test was used, where applicable, for the categorical variables. A 95% confidence interval and a statistically significant *p*-value < 0.05 were considered for all these results. 

## 5. Conclusions

Despite the limitations indicated above, the data collected show how the use of antibiotic-coated intramedullary nails seem to lead to a reduction in the infection rate in open fractures, especially when there are comorbidities that expose the patient to high infectious risk. This is not the case in closed fractures where there are insufficient data to determine whether there is an advantage in the use of gentamicin-coated nails in closed fractures. Nonunion cases are high infectious risk surgeries; gentamicin coating could protect the implant during reconstructive surgery.

These data, combined with the high safety profile of antibiotic-coated nails, suggest the use of this kind of implant in the cases mentioned above. Further data should be gathered to strengthen these results and extend the indications.

## Figures and Tables

**Table 1:** Articles included into the study. N—number of patients included into the study; FRI—Fracture-Related Infection; SSI—Surgical Site Infection, NR—Not Reported.

Author	Year	Type of Study	N	Age (Mean)	Sex (F)	Comorbidities	Multiple Trauma	Fracture Fixation Group	Revision Surgery Group	Outcome, FRI	Outcome, Superficial SSI	Outcome, Complete Bone Healing	Outcome, Partial Bone Healing	Outcome, Nonunion	Follow-Up (Month)
Fuchs et al.	2007	case report	1	17.00	0	NR	1	1	0	0	0	1	0	0	1
Raschke et al.	2010	case report	1	17.00	0	NR	1	1	0	0	0	1	0	0	13
Fuchs et al.	2011	case series	19	47.70	8	diabetes (1) smoking (2)	10	19	0	0	0	11	8	0	6
Metsemaker et al.	2015	case series	16	48.30	5	NR	5	11	5	0	0	12	0	4	18
Schmidmaier et al.	2017	cohort study	99	46.60	26	smoker (31)	12	68	31	6	8	63	11	4	18
Moghaddam et al.	2019	case series	36	46.20	8	diabetes (8) alcohol (22) smoker (11)	NR	0	36	3	4	29	0	7	12
Pinto et al.	2019	prospective case-control study	14	35.07	NR	NR	NR	14	0	0	0	10	4	0	6
Vicenti et al.	2019	case series	17	41.12	6	NR	10	0	17	0	0	17	0	0	12

**Table 2:** Preoperative and postoperative results of Fracture Fixation Group. GA—Gustilo Anderson classification; FRI—Fracture-Related Infections; SSI—Surgical Site Infection.

FF Group	n	%
Total	114	-
Closed fracture	42	36.84%
GA I	20	17.54%
GA II	23	20.17%
GA IIIa	5	4.39%
GA IIIb	12	10.53%
GA IIIc	7	6.14%
GA III und	28	24.56%
Available at last FU	105	92.10%
FRI	4	3.81%
Closed	1	0.95%
I	-	-
II	-	-
III	3	2.86%
Superficial SSI	3	2.86%
Wound Healing	1	0.95%
Dynamization	25	23.81%
Reoperation	8	7.62%
Bone healed	80	76.19%
Bone partially healed	19	18.09%
Nonunion at last FU	6	5.71%
Other complication	2	1.91%
Follow-up (month)	14.06 ± 7.06	-

**Table 3:** Preoperative and postoperative results of the Revision Surgery Group. GA—Gustilo Anderson classification; FRI—Fracture-Related Infections; SSI—Surgical Site Infection.

RS Group	n	%
Total	89	-
Nonunion	64	71.91%
Infected Nonunion	25	28.09%
Previous Closed Fractures	27	30.33%
Previous Open Fractures	62	69.66%
GA I	3	3.37%
GA II	24	26.96%
GA IIIa	4	4.49%
GA IIIb	6	6.74%
GA IIIc	3	3.37%
GA III, not specified	15	16.85%
Unknown	7	7.86%
Available at last FU	77	86.52%
FRI	5	6.49%
FRI in nonunion patients	1	
FRI in infected nonunions	4	
Superficial SSI	9	11.69%
Wound Healing Disorders	0	-
Dynamization	2	2.60%
Reoperation	6	7.79%
Bone healed	64	83.12%
Bone partially healed	4	5.19%
Nonunion at last FU	9	11.69%
Follow-up (month)	13.87 ± 3.46	-
